# Impact of prescription drug monitoring program mandate on postoperative opioid prescriptions in children

**DOI:** 10.1007/s00383-020-04846-2

**Published:** 2021-01-12

**Authors:** Christina M. Theodorou, Jordan E. Jackson, Ganesh Rajasekar, Miriam Nuño, Kaeli J. Yamashiro, Diana L. Farmer, Shinjiro Hirose, Erin G. Brown

**Affiliations:** 1 Department of Pediatric General, Thoracic, and Fetal Surgery, University of California Davis Medical Center, 2335 Stockton Blvd, Room 5107, Sacramento, CA 95817 USA; 2 Division of Biostatistics, Department of Public Health Sciences, University of California Davis, Sacramento, USA

**Keywords:** Pediatric, Opioid epidemic, Prescription drug monitoring program (PDMP), Interrupted time series (ITS)

## Abstract

**Purpose:**

Prescription drug monitoring programs (PDMPs) have been established to combat the opioid epidemic, but there is no data on their efficacy in children. We hypothesized that a statewide PDMP mandate would be associated with fewer opioid prescriptions in pediatric surgical patients.

**Methods:**

Patients < 18 undergoing inguinal hernia repair, orchiopexy, orchiectomy, appendectomy, or cholecystectomy at a tertiary children’s hospital were included. The primary outcome, discharge opioid prescription, was compared for 10 months pre-PDMP (*n* = 158) to 10 months post-PDMP (*n* = 228). Interrupted time series analysis was performed to determine the effect of the PDMP on opioid prescribing.

**Results:**

Over the 20-month study period, there was an overall decrease in the rate of opioid prescriptions per month (− 3.6% change, *p* < 0.001). On interrupted time series analysis, PDMP implementation was not associated with a significant decrease in the monthly rate of opioid prescriptions (1.27% change post-PDMP, *p* = 0.4). However, PDMP implementation was associated with a reduction in opioid prescriptions of greater than 5 days’ supply (− 2.7% per month, *p* = 0.03).

**Conclusion:**

Opioid prescriptions declined in pediatric surgical patients over the study time period. State-wide PDMP implementation was associated with a reduction in postoperative opioid prescriptions of more than 5 days’ duration.

## Introduction

The opioid epidemic is a major public health challenge facing physicians, patients, and the general public in the United States. From 1999–2016, nearly 9000 children and adolescents died in the United States from opioid overdose [1]. Alarmingly, a survey of 8888 high school seniors reported that 7% of them had used opioids for nonmedical uses in the past year, with 40% of those opioids being obtained from their own leftover prescriptions at home [2]. These facts have prompted the surgical community to examine opioid prescribing practices, with multiple studies finding excessive amounts of opioids prescribed to postoperative patients at the time of discharge [3, 4]. Although pediatric surgeons have been found to prescribe less opioids than general surgeons for the same procedure [5], there are no standardized prescribing patterns and practices vary widely [4, 6, 7]. Opioid prescriptions have been associated with higher rates of Emergency Department (ED) visits for pain and constipation [8, 9]. Families do not commonly receive instructions on disposing leftover opioids and many remain in the household [10]. Of particular concern is a recent study showing that 5% of pediatric patients continue to use opioids three to 6 months postoperatively [11].

Nationwide efforts have been made to address these problems. Educational interventions have been implemented at a number of institutions with resulting decrease in opioid prescriptions to postoperative patients [12–14]. This has not been shown to result in worse pain control in pediatric surgical patients [15]. Simply reducing the default number of pills that autopopulates in the Electronic Medical Record (EMR) when writing an opioid prescription is another intervention that was shown to reduce the number of opioid pills prescribed [16]. Nearly every state in the US now uses prescription drug monitoring programs (PDMP) to track opioid prescriptions. The state of California created a PDMP called the Controlled Substance Utilization Review and Evaluation System (CURES) which is a database of all Schedule II, III, and IV controlled substance prescriptions dispensed to patients in the state. In California, on October 2, 2018, a state-wide mandate was issued requiring consultation of the CURES database to evaluate for other controlled substance prescriptions a patient may have received. This mandate applied to any surgical patient who would be receiving a supply of opioids for more than 5 days duration [17]. A recent observational study in New Hampshire of adult postsurgical patients following institution of mandatory PDMP consultation did not show a decrease in the rate of opioid prescription [18]. However, statewide PDMPs and pain clinic legislations have been associated with decreased rates of opioid overdose in children [19].

The effect of mandatory PDMP consultation on opioid prescriptions in pediatric surgical patients has not been studied. We hypothesized that following the PDMP mandate elective pediatric surgical patients would be prescribed less opioids on hospital discharge than before. Furthermore, we hypothesized that this decrease would not be associated with an increase in ED visits for pain, or telephone calls to the surgical team for concerns related to pain.

## Methods

### Study setting and patient selection

After Institutional Review Board approval was obtained, a retrospective chart review was performed to identify patients younger than 18 years old undergoing inguinal hernia repair, orchiopexy, orchiectomy, laparoscopic appendectomy, or laparoscopic cholecystectomy with a postoperative length of stay (LOS) ≤ 24 h at a single tertiary children’s hospital between 12/1/2017 and 7/31/2019. Patients were identified by Current Procedure Terminology (CPT) code. These surgeries were chosen as they are among the most common surgeries performed in children [20]. Exclusion criteria were age > 18 years old, postoperative LOS > 24 h, if they underwent an additional simultaneous surgical procedure other than those listed above or received any opioid prescription within the 3 months prior to surgery. Cases were categorized as acute or elective procedures. Elective cases were defined as cases scheduled in clinic preoperatively, with the patient admitted electively for the purpose of undergoing the surgery. Acute cases were defined as cases scheduled urgently, with the patient presenting to the emergency department for care.

These surgeries were performed by pediatric general surgeons (*n* = 6) and urologists (*n* = 8); patients were divided equally between the two departments (see Table 1). All operating surgeons have extensive experience in these surgeries with the exception of appendectomy and cholecystectomy, which were performed exclusively by pediatric general surgeons. During the study period, there was no standardized protocol of operative techniques for the included procedures. Postoperative care was determined by the operative surgeon. Patients received standardized discharge instructions on the use of over-the-counter pain medication for postoperative pain, including instructions on the use of acetaminophen and ibuprofen.

**Table 1:** Baseline patient characteristics

	Pre-PDMP *n* = 158	Post-PDMP *n* = 228	*p* value
Age: median (IQR)	8.2 (2.8–11.5)	8.1 (2.5–12.3)	0.69
Male: *n* (%)	126 (79.8%)	170 (74.6%)	0.24
Surgical department: *n* (%)			0.93
Pediatric surgery Pediatric urology	88 (55.7%) 70 (44.3%)	128 (56.1%) 100 (43.9%)	
Postoperative LOS: median hours (IQR)	2.6 (2.1–9.1)	2.6 (1.9–5.5)	0.09
Surgical procedure: *n* (%)			0.50
Group 1: appendectomy, cholecystectomy Group 2: inguinal/testicular procedures^a^	68 (43.0) 90 (57.0)	106 (46.5) 122 (53.5)	
Acute procedure: *n* (%)	64 (40.5)	102 (44.7)	0.41

*PDMP* Prescription Drug Monitoring Program, *IQR* interquartile range, *LOS* length of stay

^a^Inguinal/testicular procedures include inguinal hernia repair, hydrocelectomy, orchiopexy, orchiectomy

### Intervention

On October 2, 2018, the state of California issued a mandate aimed at decreasing opioid prescriptions that requires providers to consult the CURES database prior to prescribing more than a 5-day supply of opioid medications to any postsurgical patient, including children and adults. The CURES database serves as California’s state-wide prescription drug monitoring program (PDMP) and requires a separate password-protected log-in for providers. At our institution, an EMR pop-up window was added, which appears for any discharge opioid prescription ordered regardless of opioid duration. The EMR pop-up window required documentation that a CURES database consultation had been performed for patients receiving greater than 5 days’ worth of opioids or documentation of a CURES exemption, such as an opioid prescription to a postoperative patient for less than 5 days’ duration. Thus, any provider entering any discharge opioid prescription into the EMR must address this pop-up alert before continuing with the prescription, regardless of the duration of the opioid prescription.

### Data collection

Charts were reviewed for the 10 months before the PDMP mandate (12/1/2017–10/1/2018) and 10 months after the PDMP mandate (10/2/2018–7/31/2019). Data collected from medical charts included demographic data, hospital and postoperative length of stay, length of surgery, surgery performed, primary surgeon, surgical department, discharge prescriptions for opioids and nonopioid pain medications, written discharge instructions, and level of provider ordering the prescription. Information was recorded on the opioid prescribed, dose, directions for use, and total amount prescribed. The following information was recorded for postdischarge clinical encounters for pain control: any phone calls regarding postoperative pain or pain medications, any unscheduled clinic visits for pain, any emergency department visits for pain, and any readmissions within 30 days of surgery. Our primary outcome was opioid prescription provided on discharge. This was analyzed in several ways: by proportion of patients receiving an opioid prescription on discharge, by duration of opioid prescription received, and by proportion of patients receiving less than 5 days’ supply of opioids on discharge. Our secondary outcomes were postdischarge clinical encounters for pain control (phone calls, unscheduled clinic visits, ED visits, or readmissions).

### Statistical analysis

The data were compared using chi-square and Fisher’s exact test for categorical data and Mann–Whitney *U* test or Kruskal–Wallis test for continuous data. Multivariable logistic regression was performed to identify factors associated with receiving an opioid prescription. Interrupted time series (ITS) analyses were performed to assess the effect of the PDMP implementation on opioid prescription rates by month. A major strength of this approach is its ability to distinguish the effect of an intervention from secular change, which is change that would have occurred over time even in the absence of the intervention. The estimation of the intervention effect is determined by comparing the trend in the outcome after the intervention to the existing trend in the preintervention period. This statistical analysis can determine if an intervention was associated with a significant change that was not ongoing prior to the intervention. All tests conducted were two-sided, and the values were considered statistically significant at the level of *p* < 0.05. Analyses were conducted using statistical software (SAS, version 9.45; SAS Institute Inc).

## Results

### Overall results

A total of 386 patients were identified, 158 patients in the pre-PDMP period and 228 patients in the post-PDMP period. The two cohorts were similar in their demographic makeup and in the proportion of children undergoing different surgical procedures (Table 1). The median age in the pre-PDMP period was 8.2 years old (IQR 2.8–11.5) and 8.1 years old (IQR 2.5–12.3) in the post-PDMP period (*p* = 0.69). Most patients were males (79.8% pre-PDMP, 74.6% post-PDMP, *p* = 0.24). All cases were performed by pediatric surgery or urology; proportion of cases performed by each was unchanged in the two cohorts (55.7% pediatric surgery pre-PDMP, 56.1% post-PDMP, *p* = 0.93). The median postoperative LOS was 2.6 h in both the pre-PDMP (IQR 2.1–9.1) and post-PDMP cohorts (IQR 1.9–5.5, *p* = 0.09). Acute cases made up 40.5% of the pre-PDMP cohort and 44.7% of the post-PDMP cohort (*p* = 0.41).

### Opioid prescriptions

In the pre-PDMP group, 32.3% of patients received an opioid prescription on discharge, compared to 15.8% of patients in the post-PDMP group (*p* < 0.001). On subgroup analysis, this decrease was only significant among elective surgical patients (48.9% of patients pre-PDMP, 20.6% post-PDMP, *p* < 0.001) with no significant change seen in acute surgical patients (7.8% pre-PDMP, 9.8% post-PDMP, *p* = 0.78). Of patients receiving opioids, patients were prescribed a median supply of 5.0 days (IQR 4.8–5.7 days) worth of opioid medications in the pre-PDMP period and a median of 4.3 days (IQR 2.3–5.2 days) in the post-PDMP period (*p* = 0.02). The proportion of patients receiving greater than a 5-day supply of opioids on discharge decreased from 13.3% pre-PDMP to 4.8% post-PDMP (*p* = 0.004).

The majority of opioid prescriptions were written for pediatric urology patients (92.2% of opioid prescriptions pre-PDMP, 80.6% of opioid prescriptions post-PDMP, *p* = 0.2). The proportion of pediatric urology patients who received discharge opioid medications significantly decreased in the post-PDMP period from 67.1 to 29% (*p* < 0.001). The proportion of pediatric general surgery patients receiving opioid prescriptions did not change (4.6% pre-PDMP, 5.5% post-PDMP, *p* = 1). The majority of prescriptions were written by resident physicians (98.2% pre-PDMP, 91.7% post-PDMP, *p* = 0.3). On multivariable logistic regression, patients had decreased odds of receiving an opioid prescription in the post-PDMP period (OR 0.23, 95% CI 0.12–0.44, *p* < 0.001), and an increased odds of receiving an opioid prescription if the surgeon was a urologist (OR 52.10, 95% CI 6.39–424.25, *p* < 0.001), or if the patient was older (OR 1.31, 95% CI 1.20–1.42, *p* < 0.001), when adjusting for type of surgery and acuity (Table 2).

**Table 2:** Multivariable logistic regression predicting opioid prescription

Variable	Odds ratio	95% CI	*p* value
Time period			
Pre-PDMP Post-PDMP	Ref 0.23	– 0.12–0.44	< 0.001
Age	1.31	1.20–1.42	< 0.001
Surgical department			
Pediatric surgery Pediatric urology	Ref 52.10	– 6.39–424.25	< 0.001
Surgical procedure			
Group 1: appendectomy, cholecystectomy Group 2: inguinal/testicular procedures^a^	Ref 0.69	– 0.07–6.68	0.75
Procedure acuity			
Elective Acute	Ref 0.27	– 0.09–0.81	0.02

*PDMP* Prescription Drug Monitoring Program, *CI* confidence interval

^a^Inguinal/testicular procedures include inguinal hernia repair, hydrocelectomy, orchiopexy, orchiectomy

### Opioid prescriptions by type of surgery

The proportion of patients who received opioids following inguinal hernia repair, hydrocelectomy, orchiopexy, or orchiectomy decreased in the post-PDMP period (Table 3). The proportion of patients who received opioids following appendectomy or cholecystectomy did not change.

**Table 3:** Patients receiving opioid prescriptions by type of surgery

Type of surgery	Pre-PDMP Received opioids: *n* (%)	Post-PDMP Received opioids: *n* (%)	*p* value
All patients: *n* (%)	51/158 (32.3)	36/2228 (15.8%)	< 0.001*
Group 1: appendectomy, cholecystectomy	3/68 (4.4)	7/106 (6.6)	0.74
Group 2: inguinal/testicular procedures^a^	48/90 (53.5)	29/122 (23.8)	< 0.001*

*PDMP* Prescription Drug Monitoring Program

^a^Inguinal/testicular procedures include inguinal hernia repair, hydrocelectomy, orchiopexy, orchiectomy

### Interrupted time series analyses

**Fig. 1:**
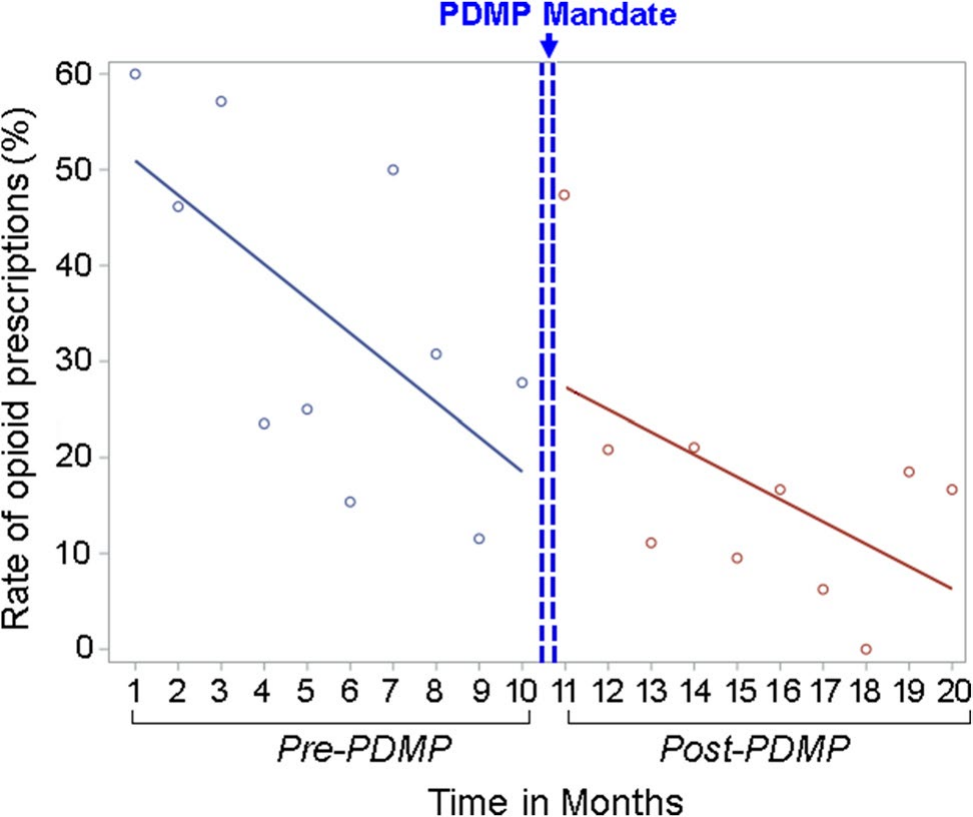
Interrupted time series analysis of effect of the PDMP mandate on overall discharge opioid prescriptions. *PDMP* Prescription Drug Monitoring Program

Interrupted time series (ITS) analysis was performed with monthly repeated measures to evaluate the association between the PDMP mandate in October 2018 with opioid prescription rates. Over the entire 20-month period, we observed an overall decrease in prescription rate per month (− 3.6% change in opioid prescription rates per month prior to PDMP mandate, 95% CI − 5.1, − 2.1, *p* < 0.001), but no significant change in opioid prescription rates per month in the post-PDMP period (11.1% change per month, 95% CI − 4.1, 26.4, *p* = 0.2). The implementation of the PDMP did not impact the rate of change of opioid prescriptions (*p* = 0.4, Fig. 1). As pediatric urology patients experienced the greatest decrease in opioid prescriptions, we repeated the ITS analysis for urology patients only. Among these patients, a significant decrease in opioid prescriptions was ongoing during the pre-PDMP period (− 4.0% per month, 95% CI − 5.7, − 2.2, *p* < 0.0001) and there continued to be a significant decrease in prescriptions during the post-PDMP period (− 4.8% per month, 95% CI − 8.9, − 0.8, *p* = 0.02). However, there was no significant difference between the slopes pre- and post-PDMP (*p* = 0.71), indicating that the PDMP mandate did not result in a decrease in opioid prescriptions among urology patients.

**Fig. 2:**
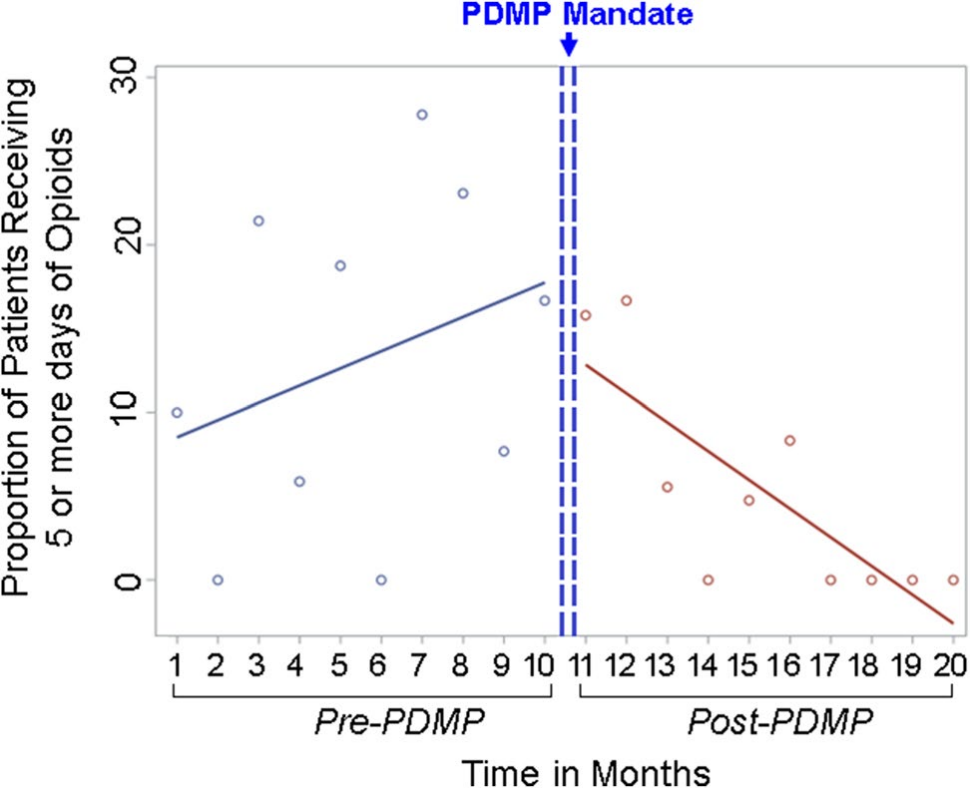
Interrupted time series analysis of effect of the PDMP mandate on discharge opioid prescriptions for greater than 5 days’ duration. *PDMP* Prescription Drug Monitoring Program

In addition, we performed an ITS analysis to investigate the effect of the PDMP intervention on the proportion of patients receiving greater than a 5-day supply of opioids on discharge, as this was the duration of opioids that required a PDMP consultation. Over the pre-PDMP period, there was no significant change in the proportion of patients receiving a greater than 5-day supply of opioids (1.0% change per month, *p* = 0.2). However, the rate of change was significantly increased in the post-PDMP period (decrease of 2.7% per month, *p* = 0.03, Fig. 2), indicating that the PDMP intervention was associated with a decrease in the rate of opioid prescriptions for a greater than 5-day supply among pediatric surgical patients.

### Postdischarge clinical encounters for pain control

There were no significant increases in phone calls for pain (8.2% pre-PDMP, 10.1% post-PDMP, *p* = 0.54), clinic visits for pain (1.8% pre-PDMP, 0.9% post-PDMP, *p* = 0.41), ED visits for pain (1.9% pre-PDMP, 2.6% post-PDMP, *p* = 0.74), or readmissions (1.9% pre-PDMP, 0.4% post-PDMP, *p* = 0.31). We repeated this analysis in the cohort of elective surgical patients to evaluate if the decrease rate of prescriptions post-PDMP was associated with increased clinical encounters for pain control, and found no significant increases in phone calls for pain (8.5% pre-PDMP, 11.1% post-PDMP, *p* = 0.65), clinic visits for pain (3.2% pre-PDMP, 0% post-PDMP, *p* = 0.08), ED visits for pain (2.1% pre-PDMP, 0.8% post-PDMP, *p* = 0.58), or readmissions (1.1% pre-PDMP, 0% post-PDMP, *p* = 0.43).

### Nonopioid pain medications

The proportion of patients receiving either acetaminophen or ibuprofen prescriptions on discharge was unchanged (7.0% pre-PDMP, 9.7% post-PDMP, *p* = 0.4), as was the proportion of patients receiving written instructions to take over-the-counter acetaminophen or ibuprofen (98.1% pre-PDMP, 100% post-PDMP, *p* = 0.07).

## Discussion

In the 10 months following implementation of a statewide prescription drug monitoring program with associated EMR pop-up window, opioid prescriptions provided to pediatric patients undergoing several common surgeries decreased by 50% compared to the 10 months before the PDMP mandate. On subgroup analysis, the decrease in opioid prescriptions was significant only for elective surgeries, with a decrease from 48.9 to 20.6% post-PDMP; opioid prescriptions following acute surgeries did not decrease but remained low at 7–9% pre- and postintervention. On interrupted time series analysis, the implementation of the PDMP mandate was not associated with a significant decrease in opioid prescriptions, suggesting that external factors not measured contributed to this overall decline in prescriptions over time. However, the primary target of the PDMP mandate was patients receiving greater than a 5-day supply of opioids postoperatively. Interrupted time series analysis showed that the PDMP mandate was associated with a significant decrease in the proportion of children receiving opioid prescriptions for a supply of greater than 5 days. It is important to note that we did not see an increase in clinical encounters related to postoperative pain, and recent literature supports that patient and family satisfaction is not compromised by this shift in practice [21]. The ongoing reduction in opioid prescriptions is significant but does not appear to be due exclusively to the PDMP mandate.

The 2018 PDMP mandate in California requires the surgeon to log in to the state-wide PDMP (CURES) database and query for other opioid prescriptions provided to their patient if they are providing greater than 5 days of opioids following surgery. As the median days of opioid medications provided was 5.0 days before the mandate and 4.3 days after the mandate, the required PDMP database query was not applicable to a large percentage of patients. However, the PDMP mandate was associated with a significant decrease in the proportion of children receiving opioid prescriptions lasting longer than 5 days, indicating a benefit of the intervention. In addition, at our institution, any patient receiving an opioid prescription in the postmandate period, regardless of duration of prescription, triggered a pop-up window in the electronic medical record and required a typed statement by the provider to proceed with the prescription. Thus, even in patients who were prescribed less than a 5-day supply of opioids, and therefore do not require PDMP consultation, the pop-up window may serve as a potential barrier to opioid prescriptions due to the additional time needed to justify not consulting the PDMP prior to providing the short-duration prescription. We believe this added step prior in the EMR likely contributes to decrease the likelihood of a surgical provider writing for an opioid medication at discharge, but in our analysis the PDMP mandate does not exclusively explain the overall reduction in opioid prescriptions.

In addition, we found widely varying rates of opioid prescriptions between the two surgical departments who performed these procedures. This difference persisted when adjusting for patient age and type of surgery and suggests potential room for improvement in our discharge practices. Provider-level practices likely contribute to opioid prescribing trends over time and represent a focus area for quality improvement. Several other groups have reported success with reducing opioid prescriptions via interventions including standardized opioid prescription protocols [12], educational interventions aimed at providers [13, 14], and reducing the default number of pills pre-specified in the EMR [16]. A no-opioid pathway following laparoscopic appendectomy has been employed with success at some institutions [21]. The significant decrease in opioid prescriptions seen in this study is likely multifactorial. Although the PDMP may serve as an important reminder to carefully consider whether opioid prescription is necessary, it does not entirely account for the decline in prescriptions. In reality, this decrease is also likely attributed to an overall change in practice as prescribers have become more aware of the dangers associated with opioid use. As most opioid prescriptions in this study were provided by resident physicians, educational interventions should include this group of providers to further reduce opioid prescriptions in pediatric patients. Attending oversight of discharge medications is critical. At our institution, we have created standardized pain medication order sets to emphasize the use of nonopioid analgesics for pediatric patients.

Unfortunately, children and adolescents are not immune to the effects of the nationwide opioid epidemic, and a large portion of recreationally used opioids in this demographic come from prior prescriptions [2]. Over 500 children and adolescents died each year over a 17-year period due to opioid overdose [1]. An analysis of a nationwide claims database of 2.7 million patients under 21 years old found that higher amounts of opioids prescribed per day was associated with increased odds of overdose [22]. Thus, it is imperative that we make every effort to reduce opioid prescriptions in this vulnerable population. Although statewide PDMPs do serve a purpose in reduction in opioid misuse and have been associated with decreased opioid overdoses in children [19] and a reduction in opioid-related deaths [23], their utility in surgical patients remains questionable. Our findings are in line with a similar study in adult surgical patients that did not find a change in overall opioid prescriptions following PDMP implementation [18], but we did demonstrate a decrease in the amount of opioid prescriptions for greater than 5 days’ duration. Further research is needed to determine how PDMPs can be best utilized to aid ongoing efforts in reduction of opioid use and misuse and which systems and educational interventions can further augment these efforts.

### Limitations

Our study has several limitations. It is a retrospective single-institution study, and thus our results may not be applicable to the general population as prescribing habits may vary at different institutions. Some patients may have been missed due to coding inaccuracies. The PDMP mandate does not require PDMP consultation if providing fewer than 5 days of opioid pain medications postoperatively; however, the EMR pop-up window provides a barrier to prescribing opioids due to the additional step in the discharge workflow if any opioids are prescribed. We believe this is barrier contributed to the lower rates of opioid prescriptions in the post-PDMP period but does not completely explain the overall decline in opioid prescription rates.

## Conclusion

Following common pediatric surgical procedures, opioid prescription rates have decreased over time. The decrease in opioid prescriptions was not associated in higher rates of postdischarge clinical encounters for poor pain control. Implementation of a state-wide prescription drug monitoring program in California was not associated with a decrease in the overall opioid prescriptions to pediatric surgical patients. However, it was associated with a decrease in the proportion of children receiving opioid prescriptions for a supply of greater than 5 days postoperatively.
